# Fitting the name or unworthy of the name?—Does the name of family firm major shareholder influence family’s egoistic behavior?

**DOI:** 10.3389/fpsyg.2022.1029380

**Published:** 2022-11-15

**Authors:** Xiaodong Yu, Huan Li, Xirong Cheng, Shize Sun

**Affiliations:** ^1^Business School, Central University of Finance and Economics, Beijing, China; ^2^School of Economics, Beijing Technology and Business University, Beijing, China

**Keywords:** family firm, major shareholder, name, egoistic behavior, Confucian symbols

## Abstract

It has been generally believed that the major shareholders of family firms are more willing to implement egoistic behaviors aimed at benefiting the family. This study analyses whether the major shareholder of the family firm whose name contains “Confucian symbols” such as benevolence, righteousness, loyalty, and kindness will reduce family self-interested behaviors as his name indicates. Using a sample consisting of all 425 family firms listed on Small and Medium Enterprise Board and Growth Enterprise Board, the result shows that the major shareholder whose name contains Confucian symbols is less likely to misappropriate corporate assets and less likely to make “family-first” personnel arrangements, meanwhile is more open to external supervision. Further mechanism testing reveals that the major shareholder whose name contains Confucian symbols also tends to choose corporate culture that reflects Confucianism. The study confirms that the name of the major shareholder is one of the factors which can affect the operation of the family business, demonstrates that different family firms have different attitudes towards family self-interest, and promotes the extant research from the “differences between family and non-family firms” level further to the “differences among family firms running by different families” level.

## Introduction

Existing studies have focused on the differences between family and non-family firms (Mullins and Schoar, 2016; Chrisman et al., 2017; Sekerci et al., 2022). Compared with non-family firms, an important task of family firms is to maintain the family’s own interests (Gómez-Mejía et al., 2007; Neff, 2015). Thus, the special goals such as enhancing family reputation (Deephouse and Jaskiewicz, 2013; Zellweger et al., 2013; Le Breton-Miller and Miller, 2020), expanding social network (Zahra, 2010; Zellweger et al., 2019), and promoting intergenerational inheritance (Berrone et al., 2012; Jaskiewicz et al., 2016; Shi et al., 2022) are given more attention because they are closely related to family interests (Souder et al., 2017). However, the achievement of these special goals is often accompanied by a loss of interest for non-family shareholders (Kellermanns et al., 2012), and as a result, it has been generally agreed that major shareholders of family firms tend to be more common in their self-interested behavior and are more likely to implement opportunistic acts in their favor (Martin et al., 2017).

However, family firms are not an entirely homogeneous group, and differences may also exist with different family firms (Daspit et al., 2021). A basic characteristic of family firms is “family-owned,” but it cannot be ignored that the families which operate the business vary from each other obviously (Yu et al., 2020). Different families have various personalities and values, and these differences are inevitably reflected in the process of family members operating family business, but existing research discussed family firms more at firm level rather than family level (Kotlar et al., 2018). From another perspective, some of the factors that influence family firms come from the family that runs the business. The characteristics of the family and the culture of the family may have an impact on family firms’ business activities such as whether to implement opportunistic behaviors, but existing research has not explored this familial difference in depth.

Recent studies have focused on the impact of manager’s name on business management (Jia et al., 2021; Kang et al., 2021), providing ideas for us to discuss the familial differences in family firms. In most countries, the name is usually given by the elders of the family. When naming the younger generation, the elders of the family often choose characters with a certain meaning according to family’s culture concept and their personal philosophy of life, so as to express their expectations, wishes, and guidance for their offspring. Thus, for the individual, the name is not only a long-term or even lifelong code and symbol, but also a reflection of the family’s ardent expectations and the elders’ earnest instruction. Since the name comes from the family elders and reflects the family culture, can the name be used as a clue to analyze the impact of family culture on family business operations, and interpret the differences within family firms based on family level? In particular, Confucianism is deeply rooted in China. Unlike the alphabet-based name structure, the Chinese name is made up of Chinese characters whose meaning can be clearly and intuitively interpreted and perceived by outsiders. Compared with the self-interested view of “family first,” Confucianism emphasizes benevolence, integrity and loyalty. Therefore, if a person’s name contains Confucian symbols such as “benevolence” (“仁”) or “righteousness” (“义”), will his self-interested motivation be suppressed accordingly when operating a family firm?

To answer these questions, this study explores the relationship between the major shareholder’s name and self-interested behavior in family firms based on the behavioral agency theory and implicit egotism theory, focusing on whether the major shareholder of the family firm whose name contains Confucian symbols such as benevolence, righteousness, loyalty, and kindness will reduce opportunistic behavior as the name indicates when running a family business, and try to look for factors that influence the opportunistic motivations of family firms based on family level. The results show that for the major shareholder of the family firm whose name contains Confucian symbols, the process of using his name reinforces the connection between Confucianism and himself. When running a family business, he is more willing to put Confucianism into practice. In particular, firstly, the major shareholder whose name contains Confucian symbols is less likely to misappropriate corporate assets. Secondly, the major shareholder whose name contains Confucian symbols is less likely to make “family-first” personnel arrangements. Thirdly, the major shareholder whose name contains Confucian symbols is more open to external supervision. The results indicate that the major shareholders of family firms take different actions in business operations due to the different meanings of their names, which demonstrates that the meaning of names can indeed influence the business philosophy of the major shareholders.

The theoretical contributions of the study are mainly in the following two aspects: first, it proves that the major shareholder’s name is one of the factors that can influence the operations of family business, incorporates the symbols that can be easily perceived by the outsiders, such as “name,” into the framework of analyzing the opportunistic motivations of family firms, explains the mechanism by which the name can influence business operations based on implicit egotism theory, and provides a new theoretical perspective for future research to analyze the business behavior of family firms’ major shareholders. Second, it focuses on the role of family culture on family business, looks for family-level factors that can influence the opportunistic motivations of family business, and extends the extant research from “differences between family and non-family firms” level further to “difference within family firms operated by different families” level.

## Literature review and hypotheses

### Implicit egotism theory

Implicit egotism emphasizes that people have an emotional or psychological preference for ideas or things which are related to themselves (Pelham et al., 2002). According to the implicit egotism theory, when an idea or a thing is intrinsically related to an individual, the idea or thing can activate this person’s subconscious positive feelings about himself, causing the individual to hold a positive attitude towards the idea or thing which he is associated with (Pelham et al., 2003). Such identification and emotional preference can exert a subtle influence on people’s decision-making in life. Existing studies have found the birthday-number preference and the name-letter effect (Jones et al., 2002; Brownlow et al., 2007), that is, people prefer the numbers in their birthdays and the letters in their names much more than other numbers and letters, and tend to choose things that are associated with their name letters or birthday numbers (Coulter and Grewal, 2014). For example, in daily life and work, people are more likely to choose to live somewhere with a name spelled similarly to their own name (Pelham and Mauricio, 2015), more likely to marry someone with the same initials in the names (Jones et al., 2004; Simonsohn, 2011a), and more likely to choose occupations with the names similar to their own or with the same initials (Simonsohn, 2011a,b). In addition, in ordinary shopping, name letters and birthday numbers can also affect consumers’ price preferences, thus affecting purchase intentions (Coulter and Grewal, 2014; Husemann-Kopetzky and Kocher, 2016), and even in baseball games, they can affect players’ performance and their position preferences on the court (Newman et al., 2009). Therefore, if an idea or a thing is closely related to the individual, people tend to accept and identify with it more, and are more willing to practice it in their daily work and life (Pelham et al., 2002; Coulter and Grewal, 2014).

### Confucian symbols and family’s egoistic behavior

Existing studies based on behavioral agency theory believe that, compared with non-family business, an important goal of the family business is to satisfy the family’s own interests (Naldi et al., 2013; Becerra et al., 2020). To achieve this goal, family firms usually maintain higher family ownership (Chu, 2011; Jiang and Peng, 2011), appoint more family executives (Verbeke and Kano, 2012; Zhang et al., 2021), and implement more stable strategies (Gómez-Mejía et al., 2018; Berrone et al., 2020), even sacrificing some economic benefits for this (Chrisman and Patel, 2012). However, to the major shareholders of a family business, the “most efficient” way to satisfy their own interests is to misappropriate the assets and resources from the firm directly (Kotlar et al., 2018). In the view of family members, the firm property is jointly owned by many shareholders, but family interests directly affect the family’s own prosperity and development. Therefore, existing studies usually believe that the major shareholders of family firms have the motivation to misappropriate corporate resources for their own use, hoping to use this as a “shortcut” to gain family interests and achieve family goals (Sacristán-Navarro et al., 2011; Chen et al., 2021).

However, due to the differences within major shareholders who run the business, family firms may have different motivation for misappropriating corporate resources to satisfy their own interests. As mentioned earlier, according to the implicit egotism theory, people generally hold a positive attitude towards ideas or things related to themselves (Pelham et al., 2003), and subconsciously this emotional preference can influence people’s decision-making (Coulter and Grewal, 2014). For example, in daily life, people tend to choose items related to their birthday numbers when shopping and are more likely to choose a community with the same initials as their own names when choosing where to live (Simonsohn, 2011a; Coulter and Grewal, 2014). Since this preference exists in people’s consciousness, it can also have an impact during the process of business operations. For the major shareholder of a family firm, if his name contains Confucian symbols such as “benevolence” and “righteousness,” he will have a more positive attitude towards Confucianism, therefore he will identify more with the business strategies related to “benevolence” and “righteousness” and will try to implement the business behaviors related to “benevolence” and “righteousness” in the process of running the business. In the circumstances, the major shareholder of a family firm pays more attention to the interests of non-family shareholders and no longer blindly seize interests for the family. As “misappropriate corporate resources for their own use” benefits the family but violates the interests of non-family shareholders, the major shareholder of a family firm who is identified with Confucianism such as “benevolence” and “righteousness,” is less likely to implement such behavior. Based on this, we propose Hypothesis 1.

*Hypothesis 1*: The major shareholder whose name contains Confucian symbols is less likely to misappropriate corporate assets.

Meanwhile, existing studies based on behavioral agency theory show that, driven by the motivation of satisfying family interests, family firms are inclined to arrange family members to work in the business (Jaskiewicz and Luchak, 2013; Basco et al., 2019). On the one hand, due to the connection of blood or marriage ties, the relationship between family members is usually closer and the interest is more consistent. Therefore, placing family members in key positions usually helps to achieve the family’s unique business goals (Jones et al., 2008; Casillas et al., 2019). On the other hand, holding a prominent position in a business often means favorable economic treatment and high social status. Therefore, leaving such opportunities to family members can better satisfy the altruistic complex among family members (Karra et al., 2006; Madison et al., 2021). However, such “nepotism” behavior inevitably has a negative impact on family business operations (Dyer et al., 2013; Leitterstorf and Rau, 2014). For example, it has been noted that many family members hold key positions in the family business just because of their family members’ identification, rather than qualified management skills (Chrisman and Patel, 2012). And if there are too many family members in a family business, it can make the family business overly focused on special business goals such as maintaining the family’s own interests, which are not conducive to the acquisition of economic interests (Stockmans et al., 2010). Therefore, existing studies generally believe that although arranging family members in key positions satisfies the family’s own interests, it is undoubtedly detrimental to the interests of non-family shareholders (Cannella et al., 2015).

However, the level of motivation to prioritize the family’s own interests may not be the same for different major shareholders. Based on the implicit egotism theory, if a person’s name contains Confucian symbols such as benevolence, integrity, and loyalty, then in the process of using his name for a long time, the person continues to strengthen his connection with Confucian symbols, identifies more with Confucian culture and is more willing to practice Confucian values. As mentioned above, arranging family members to participate in business operations, especially in key positions, although helpful to the realization of the family’s goals, can also be detrimental to the economic interests of the business and thus to the rights and interests of non-family shareholders (Gómez-Mejía et al., 2007; Ashraf et al., 2020). Therefore, if the major shareholder of a family firm whose name contains Confucian symbols identifies more with Confucian concepts such as benevolence, integrity, and loyalty, then he also pays more attention to practicing the above concepts in the process of running a family business, placing more emphasis on protecting the interests of non-family shareholders rather than blindly making personnel arrangements for family interests. In other words, the imprint of Confucian symbols makes the major shareholders of the family business incline to the latter in the balance between the egoistic behavior of chasing the family’s own interests and the “pursuit of great harmony” to protect the interests of all shareholders. Based on this, we propose Hypothesis 2.

*Hypothesis 2*: The major shareholder whose name contains Confucian symbols is less likely to make “family-first” personnel arrangements, that is, less likely to appoint family members to critical positions such as CEO.

It is precisely considering that the major shareholders of family firms generally have the motivation to give priority to satisfying their own interests, so appointing independent directors and strengthening external supervision become important ways to protect the interests of non-family shareholders (Anderson and Reeb, 2004; An and Zhang, 2013). As independent directors and other external supervision are usually unable to share the benefits derived from the self-interested behavior of major shareholders, the starting point of independent directors’ decision-making is often “maximization of corporate benefits” rather than “maximization of family interests.” However, for major shareholders, because the independent directors and other external supervision do not make decisions based on the family’s standpoint, therefore, the stronger the external supervision is, the more difficult it is for the major family shareholders to achieve the family’s special business goals and satisfy family’s own interests (Cuadrado-Ballesteros et al., 2015). As a result, it has been argued that, in order to strengthen family control and achieve family goals, family firms usually have a strong resistance to external supervision—reducing the proportion of independent directors as much as possible and weakening the power of external supervision (Anderson and Reeb, 2003).

However, not all major shareholders of family firms are equally resistant to external supervision. As mentioned earlier, based on the implicit egotism theory, people generally hold a more positive attitude towards things they are associated with (Pelham et al., 2003), and such positive attitude will further influence people’s decision-making imperceptibly (Coulter and Grewal, 2014). For the major shareholder with Confucian symbols in his name, as his name contains Confucian symbols, there is a close connection between himself and Confucianism, which makes the major shareholder more likely to recognize Confucianism and more willing to practice it in their daily work and life. This attitude is also reflected in the process of business operations. Because of focusing on practicing Confucianism such as “benevolence” and “righteousness,” the major shareholder whose name contains Confucian symbols is less likely to implement opportunistic behaviors aimed at benefiting the family. As mentioned earlier, the purpose of establishing external supervision, such as independent directors, is to restrict the opportunistic behaviors of the major shareholders of family firms (Anderson and Reeb, 2004; An and Zhang, 2013), and the reason why family firms exclude independent directors is also because external supervisory forces, such as independent directors, can prevent the major shareholders of family firms from implementing self-interested behaviors (Cuadrado-Ballesteros et al., 2015). If the major shareholder of a family firm with Confucian symbols is less likely to engage in opportunistic behaviors due to their greater acceptance of Confucianism, the psychology of reducing the proportion of independent directors and excluding external supervision will be correspondingly weaker. Based on this, we propose Hypothesis 3.

*Hypothesis 3*: The major shareholder whose name contains Confucian symbols is more open to external supervision, that is, the proportion of independent directors is higher in these firms.

## Research method

### Sample

The initial sample for this study consists of all family firms listed on the Small & Medium Enterprise Board and Growth Enterprise Board of the Shenzhen Stock Exchange of China. We use data from the Small & Medium Enterprise Board and Growth Enterprise Board because most firms listed on the Main Board are either state-owned or have strong government backgrounds, making them unsuitable for this study. While firms listed on the Small & Medium Enterprise Board and Growth Enterprise Board are less influenced by the government in their growth and development process (Yu et al., 2020). Therefore, the selection of family firms listed on the Small & Medium Enterprise Board and Growth Enterprise Board is more conducive to fulfilling the objectives of this study.

Concerning the criteria of existing studies, the study defines the family firm in a multifaceted approach based on family ownership and involvement (Gómez-Mejía et al., 2018; Kotlar et al., 2018). The “family firm” needs to meet three requirements: firstly, the actual controller of the firm is family members; secondly, the family owns at least 20% of firm shares; thirdly, at least one additional family member related to the actual controller participate in the operation of the firm. The China Securities Regulatory Commission defines a family member as “participating in the business” if that family member holds at least one of the three following positions in the firm: (a) director, (b) supervisor, and/or (c) executive in the business. In other words, if the family member holds one or more positions of director, supervisor, and executive, the family member is deemed to participate in the operation of the business.

Listed firms are required to disclose their controlling shareholders and actual controllers year by year through public materials such as annual reports, from which this study defines the major shareholder of the firm. As for whether the relatives of the major shareholder participate in the operation of the business, the firm is required to disclose the personal information of the major shareholder and the major shareholder’s relatives’ information and position in the firm in the prospectus during the initial public offering (IPO). After listing, the firm must disclose the identity and background of all directors, supervisors, and executives who join or leave the firm each year in the resolutions of the board of directors, the resolutions of the board of supervisors, the annual report of the firm, and other public materials, explaining whether they have a kinship with the major shareholder. Accordingly, we hand-collected data regarding the positions of the major shareholder’s relatives of each listed firm at the time of the IPO, updating them year-by-year by examining the materials mentioned above, as a source of the basis for judging whether the firm belongs to a family firm.

Considering that in some cases, although the major shareholder is the actual controller in the family firm, he is not involved in the operation of the business. Therefore, in this case, even if the major shareholder has an opportunistic motivation, he cannot be manifested in the operation of the business. Thus, this study excludes firms in which the chairman and general manager are not held by the major shareholder or the family members. At the same time, in some firms, the major shareholders have changed their names, especially after reaching adulthood. In order to avoid the interference of “acquired” and “artificial” name changes on the results, this study excludes family firms in which the major shareholder has a “former name.” In addition, this study also excludes the “cross-family” change of the actual controllers after listing, that is, the family firm with “non-intra-family” changes in major shareholders.

The name of the major shareholder, corporate governance, and financial data involved in this study are from the China Stock Market & Accounting Research (CSMAR) database. The Small & Medium Enterprise Board and Growth Enterprise Board of the Shenzhen Stock Exchange were established in 2004 and 2009 respectively, so data were collected from 2004 onward. While in 2018, the general market decline caused thousands of listed firms to change their actual controllers (Yu et al., 2020), thus the time span of the study sample is from 2004 to 2017. The final sample consists of 2,998 observed values of 425 families.

### Variable definition and measurement

#### Independent variable

##### Confucian symbols

The theoretical and academic circles have not given strict and clear selection criteria for which kind of name should be regarded as “name that contains Confucian symbols.” Therefore, this study uses a variety of approaches to select the characters or words that reflect the core ideas of Confucianism in order to summarize the connotations of Confucian symbols accurately and comprehensively. Specifically, the following three approaches are adopted.

The first method is based on the frequency of characters in the Analects of Confucius. As the most important work of Confucian, the Analects of Confucius embodies Confucius’ political propositions, moral concepts, and educational principles. Therefore, the more frequently a character appears in the Analects of Confucius, the closer it is to the core views of Confucius and the Confucian, and the more it conforms to the selection criteria of Confucian symbols. Therefore, the study counts the occurrence frequency of each character in the Analects of Confucius. Considering that the Analects of Confucius is a collection of Confucius’ quotations compiled by Confucius’ disciples and their further disciples, there are a large number of personal pronouns such as “self” (“子”), “I” (“吾”), “Confucius” (“孔”), adverbs such as “but” (“可”), “not” (“不”), “must” (“必”), prepositions such as “at” (“于”), “as” (“以”), “and” (“与”), neutral verbs such as “say” (“曰”), “have” (“有”), “ask” (“问”), and neutral adjectives such as “small”(“小”), “big”(“大”), “below”(“下”). Therefore, after excluding the above-mentioned words, the study focuses on characters with clear meanings and ranks them in descending order according to the frequency. The sorting results are as follows: “benevolence” (“仁,” 108 times), “courtesy” (“礼,” 74 times), “goodness” (“善,” 42 times), “virtue” (“德,” 40 times), “sincerity” (“信,” 38 times). Other words such as: “virtuous” (“贤,” 25 times), “positive” (“正,” 24 times), and “filial piety” (“孝,” 19 times), appear significantly less frequently than that of “benevolence” (“仁”), “courtesy” (“礼”), “goodness” (“善”), “virtue” (“德”), “sincerity” (“信”). Therefore, based on “the frequency of characters in the Analects of Confucius,” this study selects “benevolence” (“仁”), “courtesy” (“礼”), “goodness” (“善”), “virtue” (“德”) and “sincerity” (“信”) as “Confucian symbols” that can reflect Confucianism.

The second method is based on the interpretation of “Confucianism” and “Confucian school” in authoritative dictionaries such as Xinhua Dictionary and Contemporary Chinese Dictionary. According to Xinhua Dictionary, “Confucianism” is interpreted as “Confucius’ and his disciples’ theory, which is characterized by emphasizing the practice and cultivation of basic morals such as filial piety, benevolence, righteousness, courtesy, wisdom, and sincerity.” “Confucian school” is interpreted as “a school espousing Confucius theory, which advocates that a ruler should rule with virtue and benevolence; a gentleman should treat the elders with respect, treat friends with promise; and values ethical relations.” Based on the interpretations of the two authoritative dictionaries, the study selects the overlapping parts that are emphasized in the interpretations of “Confucianism” and “Confucian School.” Among them, the interpretation of “Confucianism” can directly extract keywords including filial piety, benevolence, righteousness, courtesy, wisdom, and sincerity. In the interpretation of “Confucian School,” the terms “rule with virtue and benevolence” and “treat the elders with respect” correspond to the term “courtesy” (“礼”), “benevolence” (“仁”) and “righteousness” (“义”) in the interpretation of Confucianism. “Treat friends with promise” emphasizes “faithful in transacting business for others, sincere in intercourse with friends,” which has the same value orientation as “sincerity” (“信”). One of the most important manifestations of “valuing ethical relations” in the Chinese cultural context is “filial piety” (“孝”). Therefore, based on the overlap and intersection between the interpretation of Confucianism in authoritative dictionaries, this study selects “courtesy” (“礼”), “benevolence” (“仁”), “righteousness” (“义”), “filial piety” (“孝”) and “sincerity” (“信”) as the “Confucian symbols” that can reflect Confucianism.

The third method is based on the summary of Confucianism generalized by later scholars. Among the various summaries of Confucianism generalized by later scholars, the “five human norms” of Confucianism, which are “benevolence” (“仁”), “righteousness” (“义”), “courtesy” (“礼”), “wisdom” (“智”) and “sincerity” (“信”), have the most profound influence. In the Three-Character Canon, it is stated that “There are benevolence, righteousness, courtesy, intelligence and sincerity. They are the five human norms, which are regulated in certain terms,” which shows that the concepts of “five human norms” of Confucianism have been deeply rooted in people’s hearts and passes down from generation to generation as important cultural treasures. Moreover, the concept of the Confucian “five human norms” is not put forward overnight, but is gradually enriched and refined by Confucian scholars of different eras. Confucius first proposed “benevolence, righteousness and courtesy,” and Mencius further extended it to “benevolence, righteousness, courtesy, and wisdom,” and Dong Zhongshu of the Han Dynasty (140 B.C.) finally defined it as “benevolence, righteousness, courtesy, wisdom and sincerity.” This summary has been inherited and tested for more than two thousand years, and has been handed down to the present day, indicating that the “five human norms” of Confucianism, that is, “benevolence” (“仁”), “righteousness” (“义”), “courtesy” (“礼”), “wisdom” (“智”) and “sincerity” (“信”), is accurate and comprehensive, and has been recognized and respected by scholars for thousands of years. Therefore, based on the summary of Confucianism generalized by later scholars, this study selects “benevolence” (“仁”), “righteousness” (“义”), “courtesy” (“礼”), “wisdom” (“智”) and “sincerity” (“信”) as “Confucian symbols” that can reflect Confucianism.

We use the methods described above to measure the variable “Confucian symbols” separately, and the regression analyses are also conducted separately to ensure the robustness of the results. If the name of the major shareholder includes the above Confucian symbols, the variable “Confucian symbols” is coded as “1,” otherwise coded as “0.” It should be emphasized that in some family firms, there is more than one actual controller of the family firm. In this regard, the study identifies the most influential family member in the firm, based on the number of shares held by each controller, their position within the firm, and their seniority in the family. As mentioned above, considering that major shareholders may change their names after reaching adulthood, this study excludes family firms in which major shareholders have a “former name.” In addition, there are no major shareholders who have Confucian symbols in their surname, that is, all Confucian symbols appear in major shareholders’ given names.

#### Dependent variable

##### Misappropriation of corporate assets

According to the existing literature, major shareholders of family firms often increase “other receivables” in order to convey corporate interests to their own stakeholders, thereby “hollow out” the listed firms (Jiang et al., 2010). Therefore, the variable “misappropriation of corporate assets” is measured by calculating the proportion of other receivables in the total assets of listed firms at the end of the period, which is caused by the parent firm and other firms controlled by the listed firm (Jiang et al., 2010). The variable is presented in percentage terms, hence, the mean value of 1.372 indicating that the other receivables make up 1.372% of total assets on average.

##### CEO family identity

The variable is measured by whether the CEO of the firm is held by a family member at the end of each year (Yu et al., 2020). If a family member serves as CEO at the beginning of the year, but leaves in the middle of the year, and the successor is not a family member, it is considered that the CEO of the year is not a family member; and vice versa. “CEO family identity” is a dummy variable, and if the CEO is a family member, the variable is coded as “1,” otherwise coded as “0.” The mean value of this variable is 0.672, indicating that in 67.2% of the observations the CEO is held by a family member.

##### External supervision level

The variable “External supervision level” is measured by the proportion of independent directors in the board of directors. The original intention of the independent director system is to make independent and objective judgments on firm decisions based on the professional perspective, and to protect the rights and interests of shareholders, especially non-family shareholders (Liu et al., 2016). Thus, the higher the proportion of independent directors in a firm, the stronger the external supervision that the family firm faces. The proportion of independent directors is calculated by dividing the number of independent directors by the board size. The mean value of the variable is 0.393, indicating that in the sample, the average percentage of independent directors is 39.3%.

#### Control variables

Besides the above variables, variables such as employee number and firm age (De Cesari et al., 2016; Zulfiqar et al., 2022) are taken into account in order to control for the possible impact of firm size and history on results. Meanwhile, considering that the operating conditions of the firm may interfere with the results, we control variables such as the firm’s asset-liability ratio and return on assets (ROA) (Miller et al., 2013; Qi et al., 2022). Moreover, to control for the potential impact of family influence, the variable family ownership is also controlled (Ahn et al., 2005). Since the personal characteristics of the major shareholders may also have an impact on the results, variables such as the major shareholders’ age, gender, and education level are added as control variables. In addition, factors such as year, industry, and province are also controlled.

## Result

### Descriptive statistic and correlation results

The descriptive statistics and correlations of the dependent, independent, and control variables are shown in Table 1.

**Table 1:** Descriptive statistics and correlations.

Variables	Mean	SD	1	2	3	4	5
1. Confucian symbols	0.033	0.179	1.000				
2. Misappropriation of corporate assets	1.372	2.361	−0.054	1.000			
3. CEO family identity	0.672	0.470	−0.090	−0.103	1.000		
4. External supervision level	0.393	0.102	0.056	0.003	−0.067	1.000	
5. Firm age	3.337	2.496	0.034	0.145	−0.074	−0.047	1.000
6. Employee number	7.209	0.982	0.022	−0.008	0.010	0.011	0.252
7. Age of major shareholders	51.854	8.396	0.041	−0.045	0.044	0.082	0.079
8. ROA	0.050	0.068	−0.024	−0.059	0.009	−0.004	−0.161
9. Family ownership	0.354	0.132	0.057	−0.047	0.068	−0.107	−0.225
10. Asset-liability ratio	0.330	0.187	−0.013	0.217	−0.097	0.014	0.240
11. Education level of major shareholders	0.875	0.664	−0.025	0.114	0.070	−0.046	0.066
12. Gender of major shareholders	0.949	0.219	0.012	0.006	−0.031	−0.044	0.051
Variables	6	7	8	9	10	11	12
6. Employee number	1.000						
7. Age of major shareholders	−0.021	1.000					
8. ROA	0.019	−0.005	1.000				
9. Family ownership	0.061	0.027	0.124	1.000			
10. Asset-liability ratio	0.361	−0.073	−0.330	0.025	1.000		
11. Education level of major shareholders	0.021	−0.278	−0.016	−0.087	0.056	1.000	
12. Gender of major shareholders	0.008	0.039	0.010	0.025	−0.033	0.004	1.000

*N* = 2,998. The absolute value of correlation coefficient greater than 0.04 is significant at *p* < 0.05.

To save space, the results of classified control variables such as industry and year are no longer listed here.

The Confucian symbols are measured based on “the frequency of characters in the Analects of Confucius.”

### Regression analyses

Based on correlation analysis, regression analysis is used to test the hypotheses. Hypothesis 1 proposes that the major shareholder whose name contains Confucian symbols is less likely to misappropriate corporate assets. The results of testing “Confucian symbols” on “misappropriation of corporate assets” are presented in Table 2. When the “Confucian symbols” is measured by “the frequency of characters in the Analects of Confucius,” there is a significant negative effect between “Confucian symbols” and “misappropriation of corporate assets” (*B* = –0.778, *p* < 0.05). When measured by “the interpretation in the authoritative dictionary,” there is still a significant negative effect between “Confucian symbols” and “misappropriation of corporate assets” (*B* = -0.813, *p* < 0.05). Also, when measured by “the summary generalized by later scholars,” there is still a significant negative effect between “Confucian symbols” and “misappropriation of corporate assets” (*B* = -0.815, *p* < 0.05), providing empirical support for Hypothesis 1.

**Table 2:** The effect of Confucian symbols on misappropriation of corporate assets.

Variable	Misappropriation of corporate assets			
	Model 1	Model 2 Measurement based on “the frequency of characters in the *Analects of Confucius*”	Model 3 Measurement based on “the interpretation in the authoritative dictionary”	Model 4 Measurement based on “the summary generalized by later scholars”
Confucian symbols		−0.778* (0.343)	−0.813* (0.345)	−0.815* (0.345)
Firm age	0.204*** (0.044)	0.209*** (0.045)	0.210*** (0.045)	0.210*** (0.046)
Employee number	−0.337*** (0.074)	−0.338*** (0.073)	−0.337*** (0.074)	−0.334*** (0.073)
Age of major shareholders	0.002 (0.007)	0.003 (0.007)	0.003 (0.007)	0.003 (0.007)
ROA	2.650*** (0.631)	2.631*** (0.633)	2.598*** (0.631)	2.595*** (0.630)
Family ownership	−0.513 (0.528)	−0.515 (0.528)	−0.497 (0.522)	−0.500 (0.521)
Asset-liability ratio	2.034*** (0.334)	2.040*** (0.335)	2.017*** (0.334)	2.015*** (0.332)
Education level of major shareholders	0.175** (0.066)	0.175** (0.066)	0.176** (0.066)	0.176** (0.066)
Gender of major shareholders	0.027 (0.264)	0.031 (0.264)	0.031 (0.264)	0.032 (0.264)
Year	Y	Y	Y	Y
Industry	Y	Y	Y	Y
Province	Y	Y	Y	Y
Observations	2,998	2,998	2,998	2,998
Groups	425	425	425	425
*R* ^2^	0.1806	0.1884	0.1888	0.1889

Standard errors are in parentheses. ***p < 0.001; **p < 0.01; *p < 0.05; **+***p* < 0.10.

Hypothesis 2 proposes that the major shareholder whose name contains Confucian symbols is less likely to make “family-first” personnel arrangements, that is, less likely to appoint family members to critical positions such as CEO. The results of testing “Confucian symbols” on “CEO family identity” are presented in Table 3. When the “Confucian symbols” is measured by “the frequency of characters in the Analects of Confucius,” there is a significant negative effect between “Confucian symbols” and “CEO family identity” (*B* = –0.927, *p* < 0.05). When measured by “the interpretation in the authoritative dictionary,” there is still a significant negative effect between “Confucian symbols” and “CEO family identity” (*B* = –0.971, *p* < 0.05). Also, when measured by “the summary generalized by later scholars,” there is still a significant negative effect between “Confucian symbols” and “CEO family identity” (*B* = –0.962, *p* < 0.05), providing empirical support for Hypothesis 2.

**Table 3:** The effect of Confucian symbols on CEO family identity.

variable	CEO Family Identity			
	Model 1	Model 2 Measurement based on “the frequency of characters in the *Analects of Confucius*”	Model 3 Measurement based on “the interpretation in the authoritative dictionary”	Model 4 Measurement based on “the summary generalized by later scholars”
Confucian symbols		−0.927* (0.395)	−0.971* (0.390)	−0.962* (0.390)
Firm age	−0.370*** (0.100)	−0.365*** (0.101)	−0.361*** (0.103)	−0.360*** (0.103)
Employee number	0.299** (0.107)	0.296** (0.107)	0.303** (0.108)	0.302** (0.107)
Age of major shareholders	0.045*** (0.009)	0.047*** (0.009)	0.048*** (0.009)	0.049*** (0.009)
ROA	−1.685* (0.682)	−1.866** (0.711)	−1.870** (0.714)	−1.872** (0.715)
Family ownership	0.588 (0.741)	0.714 (0.758)	0.716 (0.760)	0.717 (0.760)
Asset-liability ratio	−1.069** (0.401)	−1.129** (0.405)	−1.120** (0.398)	−1.121** (0.397)
Education level of major shareholders	0.214+ (0.115)	0.214+ (0.115)	0.212+ (0.115)	0.212+ (0.115)
Gender of major shareholders	0.055 (0.102)	0.057 (0.099)	0.056 (0.098)	0.057 (0.098)
Year	Y	Y	Y	Y
Industry	Y	Y	Y	Y
Province	Y	Y	Y	Y
Observations	2,998	2,998	2,998	2,998
Groups	425	425	425	425
*χ* ^2^	155.84	159.43	159.66	159.64

Standard errors are in parentheses. ***p < 0.001; **p < 0.01; *p < 0.05; **+** *p* < 0.10.

Hypothesis 3 proposes that the major shareholder whose name contains Confucian symbols is more open to external supervision, that is, the proportion of independent directors is higher in the firm. The results of testing “Confucian symbols” on the “external supervision level” are presented in Table 4. When the “Confucian symbols” is measured by “the frequency of characters in the Analects of Confucius,” there is a significant negative effect between “Confucian symbols” and “external supervision level” (*B* = 0.231, *p* < 0.001). When measured by “the interpretation in the authoritative dictionary,” there is still a significant negative effect between “Confucian symbols” and “external supervision level” (*B* = 0.208, *p* < 0.001). Also, when measured by “the summary generalized by later scholars,” there is still a significant negative effect between “Confucian symbols” and “external supervision level” (*B* = 0.216, *p* < 0.001), providing empirical support for Hypothesis 3.

**Table 4:** The effect of Confucian symbols on external supervision level.

Variable	External supervision level			
	Model 1	Model 2 Measurement based on “the frequency of characters in the *Analects of Confucius*”	Model 3 Measurement based on “the interpretation in the authoritative dictionary”	Model 4 Measurement based on “the summary generalized by later scholars”
Confucian symbols		0.231*** (0.050)	0.208*** (0.055)	0.216*** (0.056)
Firm age	0.006 (0.009)	0.004 (0.009)	0.004 (0.010)	0.004 (0.010)
Employee	0.021* (0.011)	0.020* (0.011)	0.021* (0.011)	0.021* (0.011)
Age of major shareholders	0.001 (0.001)	0.001 (0.001)	0.001 (0.001)	0.001 (0.001)
ROA	−0.039 (0.075)	−0.022 (0.075)	−0.023 (0.080)	−0.023 (0.079)
Family ownership	−0.087 (0.131)	−0.089 (0.131)	−0.103 (0.144)	−0.105 (0.140)
Asset-liability ratio	−0.006 (0.043)	−0.010 (0.043)	−0.008 (0.047)	−0.009 (0.047)
Education level of major shareholders	−0.018+ (0.010)	−0.019+ (0.010)	−0.022* (0.011)	−0.022* (0.011)
Gender of major shareholders	0.023 (0.042)	0.025 (0.047)	0.023 (0.045)	0.022 (0.045)
Year	Y	Y	Y	Y
Industry	Y	Y	Y	Y
Province	Y	Y	Y	Y
Observations	2,998	2,998	2,998	2,998
Groups	425	425	425	425
*R* ^2^	0.2173	0.2218	0.2210	0.2211

Standard errors are in parentheses. ***p < 0.001; **p < 0.01; *p < 0.05; **+***p* < 0.10.

### Further analyses: mechanism test

In order to verify the internal mechanism and basic logic, the study further analyzes the relationship between the name of the major shareholder and corporate culture. The study proposes that the reason why names can affect the opportunistic motivation of major shareholders is that major shareholders whose names contain Confucian symbols continue to strengthen their connection with Confucianism in the process of using their names for a long time. That is, they become more identified with the concept of Confucianism and pay attention to practicing Confucianism, so that when running a business, the characteristics advocated by Confucianism such as benevolence, loyalty, and integrity, are reflected more, and the opportunistic behavior aimed at their own interests is reduced. If the logic holds, then corporate culture, such as mission, vision, and values, which are the most intuitive manifestation of the major shareholders’ business philosophy, are more likely to be influenced by the personal perceptions of major shareholders. Compared with the implicit and inaccessible major shareholders’ inner thoughts and ideas, corporate culture is a more intuitive reflection of the major shareholders’ business philosophy. Therefore, the study further analyzes the relationship between the names of major shareholders and family firm culture to verify the basic logic.

By consulting the firm’s official website, the study obtains materials that can reflect the corporate culture, such as the mission, vision, and values. Whether the corporate culture contains Confucian symbols is judged according to the three methods described in the previous section. If at least one of the mission, vision, and values contains Confucian symbols, the corporate culture of the family firm is deemed to embody Confucianism. Taking “the major shareholder’s name containing Confucian symbols” as the independent variable, and “the corporate culture containing Confucian symbols” as the dependent variable, the regression results are shown in Table 5. When “the major shareholder’s name containing Confucian symbols” is measured by “the frequency of characters in the Analects of Confucius,” there is a significant positive effect between the independent variable and the dependent variable (*B* = 1.953, *p* < 0.05). When measured by “the interpretation in the authoritative dictionary,” there is still a significant negative effect between the independent variable and the dependent variable (*B* = 1.997, *p* < 0.05). Also, when measured by “the summary generalized by later scholars,” there is still a significant negative effect between the independent variable and the dependent variable (*B* = 1.990, *p* < 0.05). The results show that major shareholders of family firms whose names contain Confucian symbols are more likely to choose a corporate culture that reflects Confucianism. That is, they are indeed more identified with Confucianism and are more willing to practice Confucianism when running the business, which verifies the basic logic of the study.

**Table 5:** The effect of Confucian symbols on corporate culture.

variable	Corporate culture containing Confucian symbols			
	Model 1	Model 2 Measurement based on “the frequency of characters in the *Analects of Confucius*”	Model 3 Measurement based on “the interpretation in the authoritative dictionary”	Model 4 Measurement based on “the summary generalized by later scholars”
Confucian symbols		1.953* (0.806)	1.997* (0.831)	1.990* (0.825)
Firm age	−0.101 (0.070)	−0.101 (0.070)	−0.102 (0.070)	−0.101 (0.070)
Employee number	0.230** (0.085)	0.229** (0.085)	0.234** (0.089)	0.233** (0.087)
Age of major shareholders	−0.037*** (0.009)	−0.037*** (0.009)	−0.031*** (0.008)	−0.031*** (0.009)
ROA	0.358 (0.597)	0.355 (0.599)	0.357 (0.599)	0.357 (0.598)
Family ownership	0.021*** (0.006)	0.021*** (0.006)	0.021*** (0.006)	0.021*** (0.006)
Asset-liability ratio	−0.514 (0.360)	−0.514 (0.360)	−0.513 (0.358)	−0.513 (0.359)
Education level of major shareholders	0.109 (0.086)	0.109 (0.086)	0.108 (0.086)	0.108 (0.086)
Gender of major shareholders	0.192 (0.324)	0.193 (0.324)	0.193 (0.323)	0.193 (0.323)
Year	Y	Y	Y	Y
Industry	Y	Y	Y	Y
Province	Y	Y	Y	Y
Observations	2072	2072	2072	2072
Groups	322	322	322	322
*χ* ^2^	141.90	141.92	141.97	141.95

Note: Standard errors are in parentheses. ***p < 0.001; **p < 0.01; *p < 0.05; **+***p* < 0.10.

### Robustness test

First, to exclude the interference of the definition of the family firm on the results, the study redefines the family firm according to different criteria. Existing studies generally use the shareholding ratio of family members not less than 20% as one of the criteria for judging whether a firm is a family firm. And on this basis, the study further uses no less than 10%, 15%, and 25% family shareholding ratio as the basis for judging whether the firm is a family firm (Yu et al., 2020). Based on the re-selected samples according to different criteria, the regression analysis is carried out respectively, and the results are still consistent with the original results. Due to length limitations, this study only lists regression results under the criteria of “family shareholding ratio not less than 25%,” as shown in Table 6.

**Table 6:** Results under the criteria of “family shareholding ratio not less than 25%.”

Variable	Misappropriation of corporate assets	CEO family identity	External supervision level
Confucian symbols	−0.960** (0.358)	−0.874* (0.412)	0.258*** (0.053)
Firm age	0.204*** (0.045)	−0.384*** (0.106)	0.004 (0.009)
Employee number	−0.312*** (0.075)	0.301** (0.114)	0.027* (0.011)
Age of major shareholders	0.003 (0.008)	0.058*** (0.011)	0.001 (0.001)
ROA	2.601*** (0.637)	−1.568* (0.713)	−0.039 (0.076)
Family ownership	−0.604 (0.532)	0.011 (0.008)	−0.135+ (0.080)
Asset-liability ratio	2.043*** (0.341)	−1.066* (0.436)	−0.012 (0.044)
Education level of major shareholders	0.149* (0.068)	0.173 (0.119)	−0.020+ (0.010)
Gender of major shareholders	0.072 (0.270)	0.397 (0.408)	0.039 (0.041)
Year	Y	Y	Y
Industry	Y	Y	Y
Province	Y	Y	Y
Observations	2,770	2,770	2,770
Groups	405	405	405
*R*^2^/*χ*^2^	0.1821	152.89	0.2264

Standard errors are in parentheses. ***p < 0.001; **p < 0.01; *p < 0.05; **+***p* < 0.10.

The “Confucian symbols” are measured based on “the frequency of characters in the Analects of Confucius.”

Second, considering that the proportion of major shareholders whose names contain Confucian symbols is relatively low among the sample firms, in order to eliminate the interference of this factor on the results, this study conducts the following robustness test. As mentioned above, this study uses three methods to select the characters that best represent Confucianism, and then conducts regression analysis based on the three methods. On this basis, the study combines the characters that can represent “Confucian symbols” selected by the three methods. As long as the character is selected by any of the three methods, it is deemed to represent Confucian symbols, that is, “a Chinese character is selected if meets one of the measurement criteria.” Accordingly, the study selects “benevolence” (“仁”), “courtesy” (“礼”), “goodness” (“善”), “virtue” (“德”), “sincerity” (“信”), “righteousness” (“义”), “filial piety” (“孝”), and “wisdom” (“智”), and the proportion of the major shareholder whose name contains Confucian symbols increases from 3.3 to 6.1%. The regression results remain consistent with the original results, as shown in Table 7.

**Table 7:** Results of combining three measurement methods of the Confucian symbols.

Variable	Misappropriation of corporate assets	CEO family identity	External supervision level
Confucian symbols	−0.784* (0.345)	−0.925* (0.460)	0.229*** (0.050)
Firm age	0.208*** (0.045)	−0.394*** (0.107)	0.002 (0.009)
Employee number	−0.334*** (0.074)	0.311** (0.112)	0.021+ (0.011)
Age of major shareholders	0.003 (0.008)	0.052*** (0.011)	0.000 (0.001)
ROA	2.620*** (0.635)	−1.669* (0.719)	−0.029 (0.075)
Family ownership	−0.405 (0.511)	0.918 (0.754)	−0.001 (0.001)
Asset-liability ratio	2.035*** (0.337)	−1.095* (0.432)	−0.005 (0.043)
Education level of major shareholders	0.175** (0.066)	0.202+ (0.116)	−0.019+ (0.010)
Gender of major shareholders	0.046 (0.268)	0.468 (0.407)	0.054 (0.040)
Year	Y	Y	Y
Industry	Y	Y	Y
Province	Y	Y	Y
Observations	2,998	2,998	2,998
Groups	425	425	425
*R*^2^/*χ*^2^	0.1878	159.51	0.2227

Standard errors are in parentheses. ***p < 0.001; **p < 0.01; *p < 0.05; **+***p* < 0.10.

Third, given that the external supervision is not limited to the appointment of independent directors, the study also measures the external supervision level of family firms through the shareholding ratio of institutional investors (Gao et al., 2020). Existing research shows that, compared with individual investors, institutional investors often have higher expectations on the level of corporate governance. Institutional investors are also more sensitive to the major shareholder’s self-interested behavior, which is detrimental to their own interests, and they will take measures to prevent the major shareholders (An and Zhang, 2013). Therefore, the higher the shareholding ratio of institutional investors, the higher the level of external supervision level that the family firms are subjected to. The data on the shareholding ratio of institutional investors also comes from the CSMAR database. The results are presented in Table 8. When the “Confucian symbols” is measured by “the frequency of characters in the Analects of Confucius,” there is a significant negative effect between “Confucian symbols” and “external supervision level” (*B* = 0.112, *p* < 0.001). When measured by “the interpretation in the authoritative dictionary,” there is still a significant negative effect between “Confucian symbols” and “external supervision level” (*B* = 0.122, *p* < 0.001). Also, when measured by “the summary generalized by later scholars,” there is still a significant negative effect between “Confucian symbols” and “external supervision level” (*B* = 0.125, *p* < 0.001), Hypothesis 3 is still supported.

**Table 8:** Results of using institutional investor shareholding to measure external supervision level.

Variable	External supervision level			
	Model 1	Model 2 Measurement based on “the frequency of characters in the *Analects of Confucius*”	Model 3 Measurement based on “the interpretation in the authoritative dictionary”	Model 4 Measurement based on “the summary generalized by later scholars”
Confucian symbols		0.112*** (0.022)	0.122*** (0.022)	0.125*** (0.022)
Firm age	−0.039*** (0.006)	−0.039*** (0.006)	−0.039*** (0.006)	−0.040*** (0.006)
Employee	0.003 (0.005)	0.003 (0.005)	0.003 (0.005)	0.004 (0.005)
Age of major shareholders	0.005*** (0.000)	0.005*** (0.000)	0.005*** (0.000)	0.005*** (0.000)
ROA	−0.047 (0.030)	−0.048 (0.030)	−0.049 (0.030)	−0.055+ (0.030)
Family ownership	0.118*** (0.034)	0.119*** (0.034)	0.118*** (0.035)	0.120*** (0.035)
Asset-liability ratio	−0.051** (0.018)	−0.051** (0.179)	−0.051** (0.018)	−0.054** (0.018)
Education level of major shareholders	0.006 (0.005)	0.006 (0.005)	0.006 (0.005)	0.006 (0.005)
Gender of major shareholders	−0.059*** (0.018)	−0.058*** (0.018)	−0.060*** (0.019)	−0.058*** (0.181)
Year	Y	Y	Y	Y
Industry	Y	Y	Y	Y
Province	Y	Y	Y	Y
Observations	2,998	2,998	2,998	2,998
Groups	425	425	425	425
*R* ^2^	0.3217	0.3421	0.3430	0.3433

Standard errors are in parentheses. ***p < 0.001; **p < 0.01; *p < 0.05; **+***p* < 0.10.

Fourth, although all the family firms listed on the Small and Medium Enterprise Board and Growth Enterprise Board of the Shenzhen Stock Exchange of China are selected as research sample, considering that listed family firms are only a part of family firms, to solve the possible sample selection bias, the study adopts Propensity Score Matching (PSM) for endogeneity testing. The sample is divided into two groups according to whether the name of the major shareholder contains “Confucian symbols.” Then logit regression is used to estimate the propensity scores of sample firms, the propensity scores of the treatment group and control group are matched, and the average treatment effects of the “Confucian symbols” on “misappropriation of corporate assets,” “CEO family identity,” and “external supervision level” are calculated using the matched samples. The results of the regression using the matched samples are shown in Table 9. The coefficients of the independent variable “Confucian symbols” and the three dependent variables are consistent with the results of the original test, thus verifying the reliability of the results in this study. In addition, this study also tests for possible sample self-selection using the Heckman two-stage method, and the results shows that the regression coefficient of the inverse Mills ratio (IMR) is not significant, indicating that there is no significant sample self-selection problem in the study.

**Table 9:** Results of propensity score matching.

Variable	Misappropriation of corporate assets	CEO family identity	External supervision level
Confucian symbols	−0.730* (0.341)	−0.642* (0.321)	0.224*** (0.050)
Firm age	0.217*** (0.045)	−0.199*** (0.035)	0.001 (0.009)
Employee number	−0.407*** (0.077)	0.148* (0.071)	0.026* (0.011)
Age of major shareholders	0.000 (0.007)	0.033*** (0.007)	0.000 (0.001)
ROA	2.253** (0.853)	0.548 (1.693)	−0.106 (0.104)
Family ownership	−0.005 (0.005)	0.006 (0.005)	−0.001 (0.001)
Asset-liability ratio	2.079*** (0.349)	−1.385*** (0.387)	−0.007 (0.046)
Education level of major shareholders	0.145* (0.066)	0.273*** (0.061)	−0.019+ (0.010)
Gender of major shareholders	0.038 (0.263)	−0.076 (0.253)	0.047 (0.040)
Year	Y	Y	Y
Industry	Y	Y	Y
Province	Y	Y	Y
PSM	Y	Y	Y
Observations	2,998	2,998	2,998
Groups	425	425	425
*R*^2^/*χ*^2^	0.1843	89.20	0.2245

Standard errors are in parentheses. ***p < 0.001; **p < 0.01; *p < 0.05; **+***p* < 0.10.

The “Confucian symbols” are measured based on “the frequency of characters in the Analects of Confucius.”

Finally, the study argues that the major shareholder of family firms whose name contains Confucian symbols is less likely to implement opportunistic behaviors aimed at benefiting the family. If the view holds true, such family firms tend to have better performance. Therefore, to further validate this opinion, this study analyzes the effect of major shareholder’s name on firm performance. Referring to the existing literature, the study uses Tobin’s Q to measure the firm performance (Martínez et al., 2007; Cai et al., 2012). The results in Table 10 show that when the “Confucian symbols” is measured by “the frequency of the *Analects* words,” there is a significant positive effect between “Confucian symbols” and “firm performance” (*B* = 0.532, *p* < 0.1), indicating that the family firm with major shareholder’s name containing “Confucian symbols” has better performance, which further supports the view of this study.

**Table 10:** Effect of Confucian symbols on performance.

Variable	Firm performance	
Confucian symbols		0.532+ (0.308)
Firm age	−0.019 (0.028)	−0.023 (0.028)
Employee number	−0.764^***^ (0.092)	−0.770*** (0.093)
Age of major shareholders	−0.013^+^ (0.007)	−0.012+ (0.007)
Family ownership	0.009^*^ (0.004)	0.009* (0.004)
Asset-liability ratio	−1.616^***^ (0.315)	−1.586*** (0.315)
Education level of major shareholders	0.018 (0.052)	0.024 (0.052)
Gender of major shareholders	0.067 (0.231)	0.053 (0.232)
Year	Y	Y
Industry	Y	Y
Province	Y	Y
Observations	2,998	2,998
Groups	425	425
*R* ^2^	0.5386	0.5393

Standard errors are in parentheses. ***p < 0.001; **p < 0.01; *p < 0.05; **+***p* < 0.10.

The “Confucian symbols” are measured based on “the frequency of characters in the Analects of Confucius.”

## Discussion

The findings widely support the argument that the name of the major shareholder of a family firm can subtly influence the perceptions of the major shareholder, thereby influence the family firm’s behavior and motivation. Implicit egotism theory suggests that people always hold more positive evaluations of things or concepts that are more relevant to themselves. Accordingly, this study proposes that, for the major shareholder of a family business whose name contains Confucian symbols, the use of the name over a long period reinforces the association between major shareholder and Confucianism, and the major shareholder becomes more identified with Confucian concepts such as benevolence, integrity, and loyalty. Compared with other family firms, which are more likely to implement self-interested behaviors aimed at profiting the family, the major shareholder of a family business whose name contains Confucian symbols is more likely to put Confucian concepts such as benevolence, righteousness, loyalty, and kindness into practice when running the business, and less likely to implement opportunistic behaviors such as misappropriating corporate assets, appointing family executives, and excluding external supervision, which are intended to benefit the family but affect the interests of non-family shareholders. Whether it is less misappropriation of corporate assets, less “nepotism,” or more open to external supervision, it all means that the major shareholder of the family firm pays more attention to the interests of other shareholders, especially non-family shareholders, in the process of running the business. Therefore, the proposed hypotheses confirmed in our study prove that the name of the major shareholder is indeed one of the factors affecting the behavior of the family business, bringing the name of the family member, an explicit factor, into the analysis framework of which factors can influence business operations.

### Contributions

The theoretical contributions of the study are mainly in the following two aspects: first, it proves that the major shareholder’s name is one of the factors that can influence the operations of family business, incorporates the symbols that can be easily perceived by the outsiders, such as “name,” into the framework of analyzing the opportunistic motivations of family firms, explains the mechanism by which the name can influence business operations based on implicit egotism theory, and provides a new theoretical perspective for future research to analyze the business behavior of family firms’ major shareholders. Previous studies have focused on the influence of managers’ demographic characteristics, experience backgrounds, personalities, and values on their business operations. Compared with factors such as personalities and values, which are difficult to be detected, a person’s name can be quickly and accurately accessed by outsiders. Therefore, analyzing the impact of major shareholder’s name on family business operation helps outsiders to get to know and make a judgement on the operation behavior and style of family business quickly. Also, this study uses implicit egotism theory to illustrate the intrinsic mechanism by which the name of the major shareholder influences business behavior. In previous studies, when analyzing the manager’s business philosophy, the emotional distance between the manager and the philosophy is seldom mentioned. However, this study based on the perspective of “name,” proposes and proves that the reason why manager prefers a certain business philosophy is because the philosophy is related to the manager himself in some way, which provides a new idea for further studies to analyze manager’s decision-making and behavior.

Second, it focuses on the role of family culture on family business, looks for family-level factors that can influence the opportunistic motivations of family business, and extends the extant research from “differences between family and non-family firms” level further to “difference within family firms operated by different families” level. Previous studies have suggested that family firms tend to have stronger opportunistic motivations than non-family firms due to the priority of satisfying family interests. However, this study further points out that different family firms, due to the differences in operating families, have different views on opportunistic motivations. Some families show lower opportunistic motivations because they value Confucian concepts such as benevolence and righteousness and also focus on practicing these concepts when running the business. Compared with previous studies that focused more on firm-level factors such as governance structure and business goals, this study focuses on how family-level variable affects the opportunistic motivations of family firms, which leads to a shift in the focus of existing research from “family business” to “family that runs the business,” and provides a theoretical fulcrum for future research on the topic of “what are the differences within family firms run by different families.”

### Limitations and future research

Inevitably, this study has limitations, which also offer implications for future research. First, although the study adopts three methods to measure the variable “Confucian symbols,” including “the frequency of characters in the Analects of Confucius,” “the interpretation in the authoritative dictionary,” and “the summary generalized by later scholars,” to provide a comprehensive and systematic interpretation of the meaning of the variable “Confucian symbols.” However, it is undeniable that there must be other methods for measuring the variable. Whether the results and conclusions based on other methods are still consistent with this study requires further testing.

Second, this study analyzes the effect of the major shareholder’s name on the self-interested behavior of family firms, taking only listed family firms as a sample. While future studies could choose the data of unlisted family firms to further test whether the findings of this study are generalizable. Like listed firms, unlisted firms also confront the second-tier principal-agent problem that major shareholders of family firms infringe on the interests of non-family shareholders. However, compared with listed family firms, unlisted family firms have simpler governance structures and receive less regulatory attention, making their daily operations more likely to be influenced by the personal will of the major shareholders. Given this, whether the findings of the study can be generalized to unlisted family firms, that is, whether the major shareholder whose name contains “Confucian symbols” can affect the family’s self-interested behavior in unlisted family firms, also needs to be further explored in future research.

## Data availability statement

The original contributions presented in the study are included in the article/supplementary material, further inquiries can be directed to the corresponding author.

## Author contributions

XY: made the theoretical design of this article, reviewed, and revised the manuscript. HL: drafted the manuscript. XC and SS: collected and analyzed the data. All authors contributed to the article and approved the submitted version.

## Funding

The author(s) disclosed receipt of the following financial support for the research, authorship, and/or publication of this article: National Natural Science Foundation of China (No. 72002232), Humanity and Social Science Foundation of Ministry of Education of China (No. 19YJC630207) and Outstanding Youth Project of Central University of Finance and Economics (No. QYP202106).

## Conflict of interest

The authors declare that the research was conducted in the absence of any commercial or financial relationships that could be construed as a potential conflict of interest.

## Publisher’s note

All claims expressed in this article are solely those of the authors and do not necessarily represent those of their affiliated organizations, or those of the publisher, the editors and the reviewers. Any product that may be evaluated in this article, or claim that may be made by its manufacturer, is not guaranteed or endorsed by the publisher.
